# Vasospastic angina in a chronic myeloid leukemia patient treated with nilotinib

**DOI:** 10.1186/s40959-021-00119-6

**Published:** 2021-08-27

**Authors:** Shunsuke Maruta, Kyohei Usami, Kazuko Tajiri, Masafumi Otani, Daigo Hiraya, Hiroaki Watabe, Tomoya Hoshi, Akira Sato, Masaki Ieda

**Affiliations:** Department of Cardiology, Faculty of Medicine, University of Tsukuba, 1-1-1 Tennodai, Tsukuba, Ibaraki 305-8575 Japan

**Keywords:** Chronic myeloid leukemia, Coronary angiography, Coronary circulation, Coronary spasm, Vasospastic angina, Tyrosine kinase inhibitor, Cardio-oncology, Onco-cardiology

## Abstract

**Background:**

Nilotinib, a second-generation BCR-ABL tyrosine kinase inhibitor (TKI), is highly effective in the treatment of patients with chronic myeloid leukemia (CML), despite being more vasculotoxic than older TKIs such as imatinib. Herein, we present a case of nilotinib-associated vasospastic angina confirmed by an acetylcholine spasm provocation test.

**Case presentation:**

A 62-year-old CML patient treated with 300 mg nilotinib twice daily complained of several episodes of rest angina and was hospitalized at our institution. Coronary angiography revealed no severe organic stenosis, and the acetylcholine spasm provocation test confirmed the diagnosis of vasospastic angina. Although treatment with a calcium channel blocker and nicorandil reduced the frequency of chest pain, angina symptoms continued to occur. At 10 months post discharge, the patient complained of increased frequency of angina; therefore, the nilotinib dosage was reduced to 150 mg twice daily. Consequently, the patient reported a significant improvement in chest symptoms.

**Conclusions:**

This case report highlights the potential vasculotoxic effects of nilotinib. Cardiologists and hematologists should be vigilant for coronary artery spasm as a possible vascular adverse event caused by nilotinib.

**Supplementary Information:**

The online version contains supplementary material available at 10.1186/s40959-021-00119-6.

## Background

Chronic myeloid leukemia (CML) is a myeloproliferative disorder characterized by a genetic translocation between chromosomes 9 and 22, leading to the generation of the hybrid protein BCR-ABL with tyrosine kinase activity. For most CML patients, the introduction of BCR-ABL tyrosine kinase inhibitors (TKIs) has changed this fatal disease into a manageable chronic condition [1, 2]. However, recent studies have revealed that patients treated with newer BCR-ABL TKIs show a significant increase in the incidence of vascular adverse events, including increased blood pressure, venous thrombosis, progressive atherosclerosis with coronary artery disease, and peripheral arterial obstructive disease [2, 3]. Nilotinib, a second-generation TKI, is highly effective in the treatment of CML patients both as a first-line treatment and after imatinib treatment failure; however, it is known to be more vasculotoxic than older TKIs, including imatinib [2]. Herein, we present a rare case of nilotinib-associated vasospastic angina confirmed by an acetylcholine (ACh) spasm provocation test.

## Case presentation

A 62-year-old man, who was an ex-smoker with hyperlipidemia, was diagnosed with CML in 2010. The patient was initially treated with imatinib. However, due to the appearance of a rash, the patient was switched to 300 mg nilotinib twice daily in May 2011, resulting in a major molecular response in May 2013. Subsequently, nilotinib was maintained at this dose. The patient had no history of heart disease and had not undergone any cardiovascular procedures before the start of CML therapy. In April 2019, 8 years after the initiation of nilotinib treatment, the patient complained of several episodes of rest angina, which most frequently occurred in the early morning. Resting chest pain, sometimes accompanied by cold sweats, was quickly resolved with nitrates. The patient was admitted to the hospital with suspected angina pectoris.

**Fig. 1:**
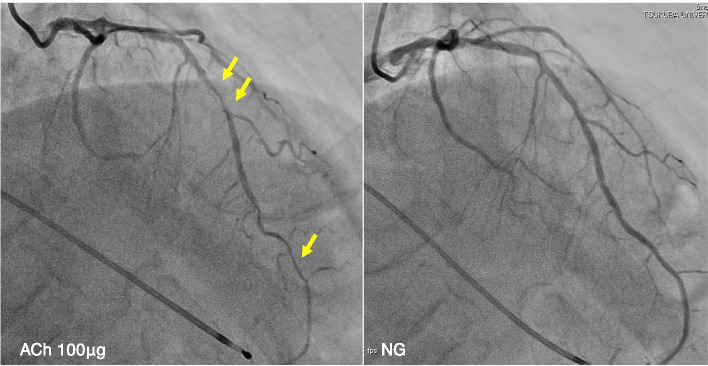
Coronary angiography findings. Left panel shows coronary spastic portion (arrows) at the mid left anterior descending artery after 100 μg of acetylcholine injection. The right panel shows no stenosis in the left coronary artery after nitroglycerin (NG) administration

**Fig. 2:**
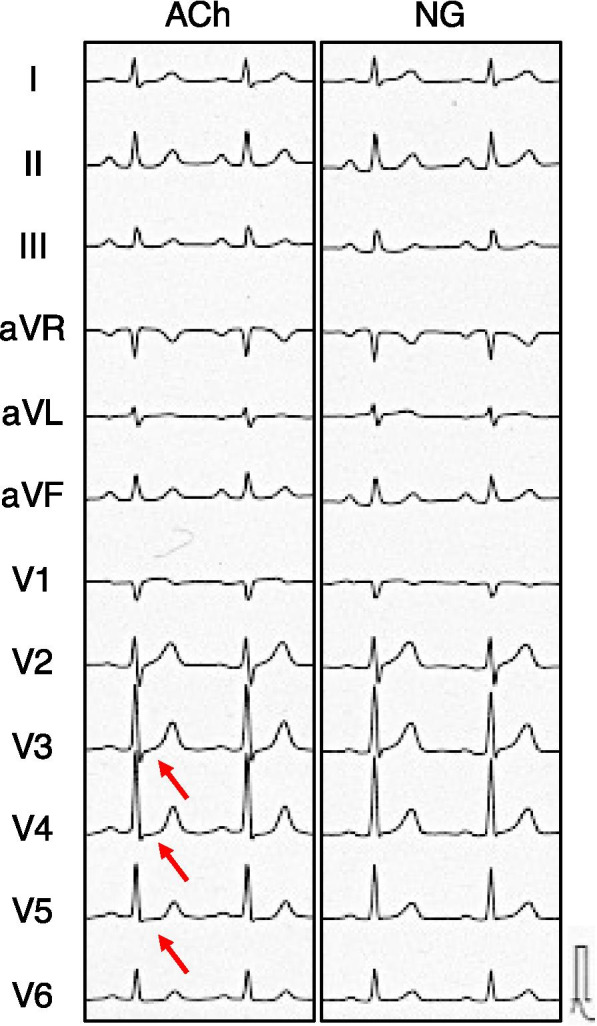
Electrocardiogram findings. Electrocardiogram recording after 100 μg acetylcholine injection (left) and after nitroglycerine (NG) injection (right). Red arrows indicate ST-segment depression

Electrocardiography (ECG) performed at the time of admission revealed no ST-segment abnormalities. No abnormal left ventricular wall motion was detected on echocardiography (Supplemental Figure 1). The patient underwent coronary angiography, which showed a 25% stenosis of the proximal right coronary artery, 50% stenosis of the proximal to mid right coronary artery, and no remarkable stenosis in the left coronary artery. Since coronary angiography revealed no severe organic stenosis, an acetylcholine spasm provocation test was subsequently performed. Intracoronary administration of 100 μg ACh into the left coronary artery resulted in 90% vasoconstriction of the mid segment of the left anterior descending artery (Fig. 1). The patient experienced severe chest pain, and ST-segment depression in the V3-V5 leads was recorded on the electrocardiogram (Fig. 2). Coronary flow, chest symptoms, and ST-segment depression on ECG promptly improved after the injection of nitroglycerin into the left coronary artery (Figs. 1 and 2).

The patient was diagnosed with vasospastic angina, and treatment with a calcium channel blocker and nicorandil was initiated. Although the administration of coronary vasodilators reduced the frequency of chest pain, angina symptoms continuously recurred (1–2 times a month). At 10 months post discharge, the patient complained of an increase in the frequency of angina; therefore, the nilotinib dosage was reduced to 150 mg twice daily. Consequently, the patient reported a significant improvement in chest symptoms (Supplemental Figure 2).

## Discussion

To date, only two cases of vasospastic angina have been reported as a side effect of BCR-ABL TKI treatment in the literature [4], and this is the first case confirmed by a spasm provocation test. On ACh provocation testing, the patient displayed typical chest pain, ischemic ECG changes, and ≥ 90% vasoconstriction on angiography, which indicated epicardial spasm, based on the criteria set by the Coronary Vasomotor Disorders International Study (COVADIS) [5] and Japanese Circulation Society (JCS) guidelines [6]. The patient showed hallmark features of vasospastic angina, including angina at rest that frequently occurred in the early morning, which was promptly resolved by administration of short-acting nitrates. The patient was treated with calcium-channel blockers, resulting in the suppression of symptoms [7].

The classic cause of ischemic heart disease is coronary atherosclerosis; however, approximately one-half of patients undergoing diagnostic coronary angiography for typical chest pain show no significant organic coronary stenosis [8]. In such cases, coronary vasomotor disorders, including vasospastic angina (epicardial coronary spasm) and/or microvascular angina, may be involved [7]. Patients with vasospastic angina usually have a favorable prognosis and good long-term survival, especially those who quit smoking, receive optimal medical therapy with calcium channel blockers, do not have multi-vessel involvement, and have no underlying obstructive coronary lesions [7]. However, these interventions first require an accurate diagnosis. Without appropriate diagnosis to guide treatment, vasospastic angina may lead to life-threatening arrhythmias and sudden cardiac death [7]. Although rarely performed in daily practice, pharmacological spasm provocation tests are recommended according to the guidelines established by the European Society of Cardiology [9], JCS [6] and COVADIS consensus [5]. In our case, the patient underwent an ACh spasm provocation test, which confirmed the diagnosis of definitive vasospastic angina. Although the administration of coronary vasodilators reduced the frequency of chest pain, the symptoms could not be completely controlled. After reducing the nilotinib dose, the patient showed significantly improved chest symptoms. Thus, this highlights the importance of establishing a diagnosis and providing appropriate treatment, thereby allowing optimal concomitant cancer treatment.

The central players involved in vasospastic angina are endothelial cells and vascular smooth muscle cells, and the interplay of these cells plays a crucial role in the adequate regulation of coronary vascular tone [7, 10]. Recent mechanistic studies investigating the effects of nilotinib on the vascular system have shown decreased endothelial cell viability and increased expression of molecular patterns related to apoptosis [11, 12]. Vascular endothelial dysfunction occurs during the aging process and can be induced by established cardiovascular risk factors such as hypertension, hyperlipidemia, diabetes, obesity, and smoking [10]. Our patient was relatively old (70 years old at the onset of angina) and an ex-smoker with hyperlipidemia. Thus, endothelial dysfunction may have been present in the background, which could be exacerbated by nilotinib and cause vasospastic angina. Many factors, including cardiovascular risk factors, can play a role in the occurrence of endothelial and smooth muscle dysfunction, the underlying mechanisms of vasomotor disorders. Consistent with our case, not only nilotinib but also various patient factors can be considered as the cause of angina in a complex manner. Recent studies have suggested that enhanced Rho-kinase activation is involved in coronary hyperreactivity [13]. However, the underlying mechanism of coronary artery spasm resulting from nilotinib treatment is unknown. Further investigations are required to elucidate whether coronary artery spasm is related to the off-target effects of nilotinib and the kinases involved.

Vascular events including cardiac and cerebral ischemic events and peripheral arterial occlusive disease have become serious clinical problems for patients receiving BCR-ABL TKIs (particularly ponatinib and nilotinib) [2, 14]. The rates of vascular adverse events in clinical trials considerably varied because the trials were not designed to assess at this point, and vascular risk factors were not properly assessed at pre- and post-treatment. After a 2-year observation period, the percentage of patients with CML developing vascular adverse events during nilotinib was reported 1–29% [15]. The mechanisms underlying the vascular toxicity of BCR-ABL TKIs remain unclear. Several clinical studies have suggested that nilotinib is associated with hyperglycemia and hypercholesterolemia [16], the major risk factors for the occurrence of atherosclerosis. In in vitro experiments, nilotinib has marked effects on vascular endothelial cells, including the induction of interleukin-1β and adhesion molecule production in association with downregulation of miR-3121-3p expression in endothelial cells [17]. Thus, nilotinib may directly affect endothelial dysfunction, resulting in ischemic vascular adverse events, such as coronary artery spasm and atherosclerotic events. Due to the high frequency of adverse vascular events associated with BCR-ABL TKIs, patients with CML considered to be at high risk based on comorbidities or TKI selection should be carefully monitored.

Coronary artery spasm due to fluoropyrimidines such as 5-fluorouracil (5-FU) and its oral form, capecitabine, is well recognized by oncologists and cardiologists [18]. The incidence of myocardial ischemia considerably varies and may be as high as 10%, depending on the dose, scheduling, and way of administration [19]. The median time from 5-FU infusion to symptom onset has been reported to be 12 h, which can occur up to 1–2 days after injection [20]. Contrary to 5-FU, the development of coronary spasm seems to require a long time due to BCR-ABL TKI administration. In the three previously reported cases of BCR-ABL TKI-induced spasm, including our case, the time from the administration to symptom onset has been reported to be 3–8 years [4] because of its different effects on the endothelium and vascular smooth muscle depending on the drugs; however, the details are still unknown.

## Conclusions

In summary, this is a rare case of nilotinib-associated vasospastic angina confirmed by the ACh spasm provocation test. Cardiologists and hematologists should be vigilant for coronary artery spasm as a possible vascular adverse event caused by nilotinib. Careful long-term follow-up should be performed, especially in patients with CML with coronary risk factors.

## Supplementary Information

**Additional file 1**

## Data Availability

The datasets used and/or analyzed during the current study are available from the corresponding author on reasonable request.

## Abbreviations

ACh: Acetylcholine
CML: Chronic myeloid leukemia
COVADIS: Coronary Vasomotor Disorders International Study
ECG: Electrocardiography
JCS: Japanese Circulation Society
TKI: Tyrosine kinase inhibitor
