# Supplementary figures and images for: Vasospastic angina in a chronic myeloid leukemia patient treated with nilotinib
Source: Cardiooncology. 2021 Aug 27;7:31. doi: 10.1186/s40959-021-00119-6 (PMC8393793; doi:10.1186/s40959-021-00119-6)

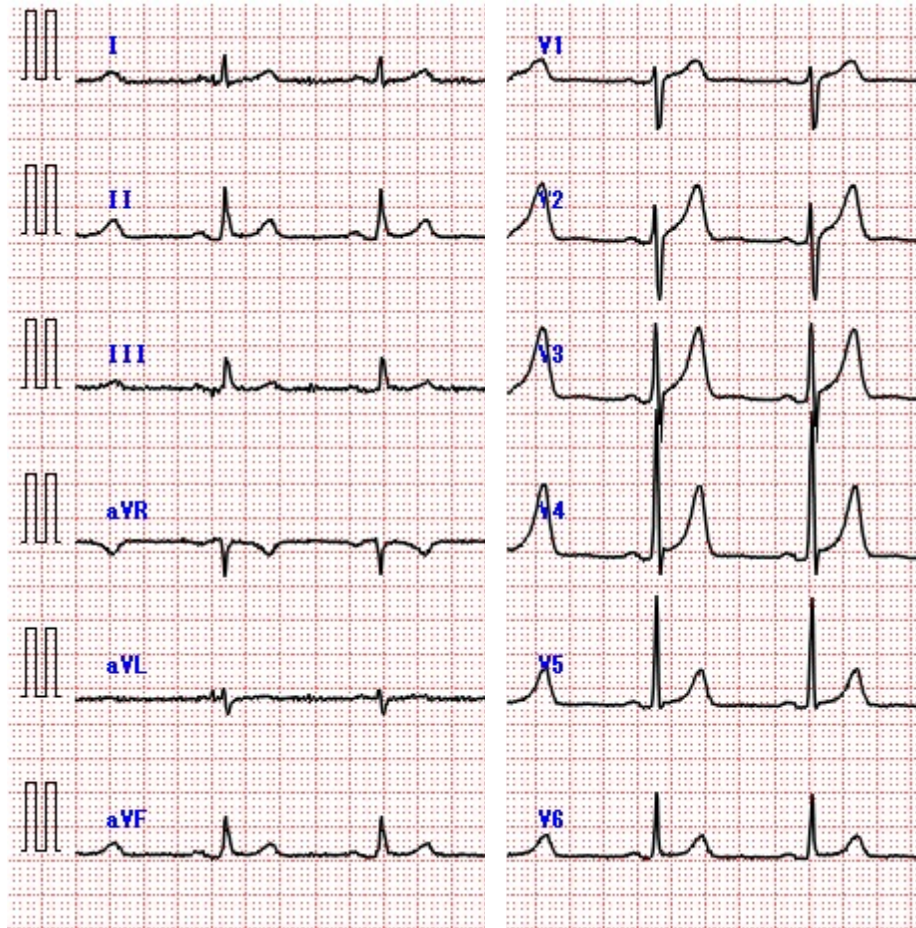

**Supplementary Figure 1. Electrocardiogram on admission**

Supplement: Supplementary file 1 — Additional file 1 [file 40959_2021_119_MOESM1_ESM.zip › Supple Fig1.pdf]

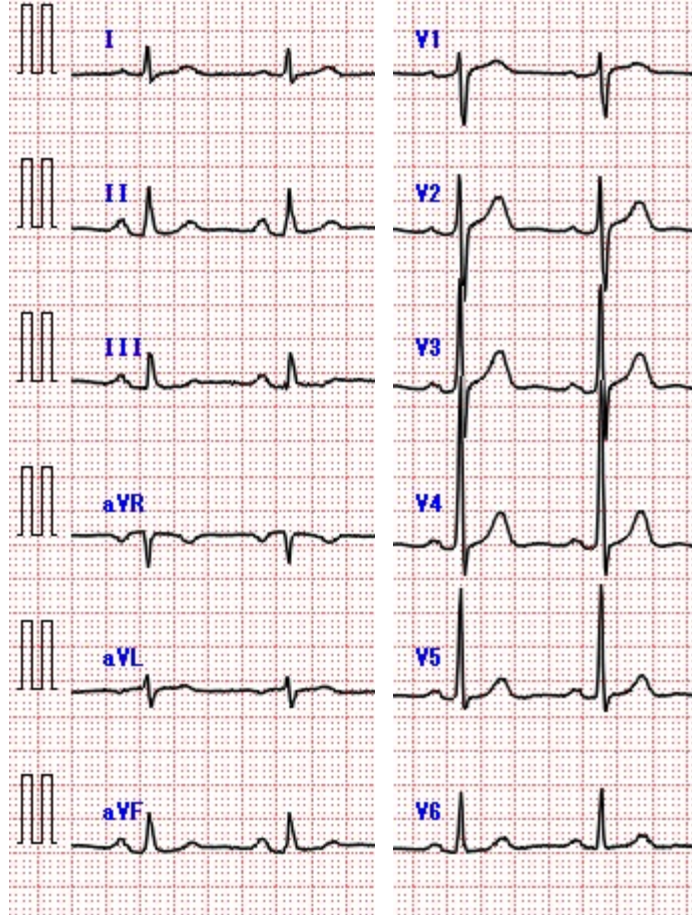

**Supplementary Figure 2. Electrocardiogram after nilotinib dose reduction**

Supplement: Supplementary file 1 — Additional file 1 [file 40959_2021_119_MOESM1_ESM.zip › Supple Fig2.pdf]
